# Molecular Mechanisms of Uterine Leiomyosarcomas: Involvement of Defect in LMP2 Expression

**DOI:** 10.4137/grsb.s470

**Published:** 2008-07-22

**Authors:** Takuma Hayashi, Yuto Shimamura, Taro Saegusa, Akiko Horiuchi, Yukihiro Kobayashi, Nobuyoshi Hiraoka, Yae Kanai, Hiroyuki Aburatani, Kenji Sano, Ikuo Konishi

**Affiliations:** 1 Dept. of Immunology and Infectious Disease, Shinshu University Graduate School of Medicine; 2 Dept. of Obstetrics and Gynecology, Shinshu University Medical School; 3 Dept. of Laboratory, Medicine, Shinshu University Hospital; 4 Pathology Division, National Cancer Center Research, Institute; 5 The Cancer System Laboratory, Research Center for Advanced Science and Technology, The University of Tokyo; 6 Japan Science and Technology Agency

**Keywords:** uterus, leiomyosarcoma, lmp2, IFN-γ signalling pathway

## Abstract

Patients with uterine leiomyosarcoma (LMS) typically present with vaginal bleeding, pain, and a pelvic mass. Typical presentations with hypercalcemia or eosinophilia have been reported. Radiographic evaluation with combined positron emission tomography/computed tomography may assist in the diagnosis and surveillance of women with uterine LMS. A recently developed risk-assessment index is highly predictive of disease-specific survival. Ovarian preservation does not appear to negatively impact outcome, and the addition of adjuvant therapy after surgical treatment does not seem to improve survival. It is noteworthy that LMP2-deficient mice exhibit spontaneous development of uterine LMS with a disease prevalence of ~37% by 12 months of age. The *LMP2* gene is transcribed from a promoter containing an interferon (IFN)-γ-response factor element; thus, the IFN-γ-signal strongly induces *LMP2* expression. Furthermore, a recent report demonstrated the loss of ability to induce LMP2 expression, which is an interferon (IFN)-γ-inducible factor, in human uterine LMS tissues and cell lines. Analysis of human uterine LMS shows somatic mutations in the IFNγ signalling pathway, thus the loss of LMP2 induction is attributable to a defect in the earliest steps of the IFN-γ signalling pathway. The discovery of an impaired key cell-signalling pathway may provide new targets for diagnostic approaches and therapeutic intervention.

## Introduction

Smooth muscle tumors (SMTs) have been traditionally divided into benign leiomyomas (LMA) and malignant leiomyosarcomas (LMS) based on cytological atypia, mitotic activity and other criteria. Uterine LMS, which are some of the most common neoplasms of the female genital tract, are relatively rare SMTs, having an estimated annual incidence of 0.64 per 100,000 women (Zaloudek and Hendrickson, 2002). They account for approximately one-third of uterine sarcomas, and are considered to be aggressive malignancies with a five-year survival rate of only 53% for tumors confined to the uterus (Gadducci et al. 1996; Nordal and Thoresen, 1997). Gynecological cancer, for instance breast cancer and endo-metrial carcinomas, are strongly promoted by female hormones, but the rate of hormone receptor expression is reported to be significantly less in human uterine LMS compared with normal USM cells. These low receptor expressions were found to not correlate with the promotion of initial disease development or with the overall survival of patients with uterine LMS; however, molecular targeting therapies against tumors have recently shown remarkable achievements (Leitao et al. 2004; Perez et al. 2004). It is noteworthy that, when adjusting for stage and mitotic count, LMS has a significantly worse prognosis than carcinosarcoma (Miettinen and Fetsch, 2006). As uterine LMS is resistant to chemotherapy and radiotherapy, and thus surgical intervention is virtually the only means of treatment for this disease (Brooks et al. 2004; Dusenbery et al. 2005; Wu et al. 2006), developing an efficient adjuvant therapy is expected to improve the prognosis of the disease. A trend towards prolonged disease-free survival is seen in patients with matrix metalloproteinase (MMP)-2-negative tumors (Bodner-Adler et al. 2004). Although typical presentations with hypercalcemia or eosinophilia have been reported, this clinical abnormality is not an initial risk factor for uterine LMS. LMP2 knockout mice exhibit a defect in proteasome function, and LMP2^−/−^ female mice are shown to develop uterine leiomyosarcomas, with a disease prevalence of 36% by 12 months of age (Van Kaer et al. 1994; Hayashi and Faustman, 2002). Furthermore, a recent report showed that the loss of LMP2 expression in human LMS tissues is probably attributable to a defect in the earliest steps of the IFN-γ-signalling pathway. Defective LMP2 expression may initiate the development of spontaneous human uterine LMS (Hayashi and Faustman, 2002; Hayashi et al. 2006). Because there is no effective therapy for unresectable uterine LMS, these findings may enable the development of diagnostics and specific molecular therapies to treat this disease.

## LMP2-Deficient Mice Exhibit Spontaneous Development of Uterine LMS

Although gynecological cancers, for instance, breast cancer and endometrial carcinomas, are stongly promoted by female hormones, the rate of hormone receptor expression is reported to be significantly less in uterine LMS than in normal uterine smooth muscle (USM). As apoptotic mechanisms have also been implicated in many human cancers, investigating the dysregulation of the expression of apoptotic and/or cell-cycle regulators in uterine LMS is required to identify molecular pathways that could possibly be important in the development of human uterine LMS. Although the significant differential expression of apoptotic and cell-cycle regulatory factors, including initiation factor, in human uterine LMS have all been reported and compared to normal USM, there exists no scientific evidence to show that abnormal expression of these factors directly correlates to the initiation and promotion of uterine LMS (Zhai et al. 1999; Miyajima et al. 2001; Raspollini et al. 2005; Alhopuro P. et al. 2005; Leiser et al. 2005; Anderson et al. 2006).

**Figure 1:**
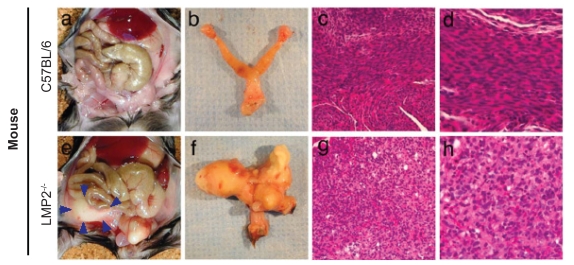
Development of uterine neoplasms in LMP2^−/−^ mice. Uterine neoplasms in LMP2^−/−^ mice. a and e, abdominal cavities of female C57BL/6 (**a**) and LMP2^−/−^ (**e**) mice, showing a uterine tumor (outlined by yellow arrowheads) in the latter. b and f, female genital organs of C57BL/6 (**b**) and LMP2^−/−^ (**f**) mice, showing a uterine neoplasm in the latter. c, d, g, h, histologycal analysis showing the normal smooth muscle cells of the uterus of C67BL/6 mice (**c** and **d**) and the abnormal cells of a leiomyosarcoma of the uterus of LMP2^−/−^ mice (**g** and **h**). c,g, × 200; d,h, × 400.

The targeted disruption of LMP2 results in the impairment of tissue- and substrate-dependent proteasome function (Van Kaer et al. 1994). LMP2^−/−^ mice were reported to be prone to the development of uterine neoplasms (Hayashi and Faustman, 2002). The percentage of mice with overt tumors increased with age after six months, with a cumulative prevalence of disease in female mice of 37% by 12 months of age and no apparent plateau at this late observation time. LMS was observed in LMP2^−/−^ female mice but not in their parental mice, C57BL/6 mice (Hayashi and Faustman, 2002) (Fig. 1). Histological examinations of LMP2^−/−^ uterine neoplasms revealed common characteristic abnormalities of uterine LMS (Fig. 1). The tumors lacked lymphoid infiltrates, which is a sign of immune recognition, and consisted of uniform elongated USM cells arranged into bundles. The nuclei of the tumor cells varied in size and shape; furthermore, mitosis was frequent. In contrast, USM cells of C57BL/6 mice were normal in appearance, and relatively few Ki-67-positive cells, the proliferating cells of solid tumors, were observed in the basal cell layer of normal USM, whereas most basal cells vividly expressed Ki-67 in LMP2^−/−^ mice (Hayashi and Faustman, 2002). These histopathological studies indicate the abnormal proliferation of LMP2^−/−^ USM cells in the basal cell layer of normal USM. In LMP2^−/−^ mice, proteasomal activity against hydrophobic and basic substrates but not acidic substrates was lower in the muscle. Furthermore, flow cytometric analysis showed no difference in the expression of MHC class I molecules. Importantly, spontaneous uterine LMS was particularly detected, but no other tumor progression was observed at high/low incidences in both male and female LMP2^−/−^ mice; therefore, LMP2 expression, rather than providing an escape from immune surveillance, seems to play an important role in the spontaneous development of uterus LMS.

## Correlation Between Defective LMP2 Expression and Human Uterine LMS

Several reports suggest that INF-γ-induced restoration of antigen-processing machinery improves anti-tumor-specific antigen CTL recognition in some patients; thus, approaches to activate this pathway may be of benefit to patients with LMP2 deficiency. Furthermore, it should be demonstrated whether human uterine LMS shows a weak expression of LMP2. The effects of IFN-γ on LMP2 expression was examined using five cell lines (Hayashi et al. 2006). LMP2 expression were not markedly induced by IFN-γ treatment in human uterine LMS cell lines, although cervical epithelial adenocarcinoma cell lines and normal human uterus smooth muscle cells underwent strong induction of LMP2 following IFN-γ treatment (Hayashi et al. 2006). Furthermore, the experiments, performed separately at several medical facilities, revealed a serious loss in the ability to induce LMP2 expression in human uterine LMS tissues in comparison with normal USM tissues located in same tissue sections: normal total, 26 cases; LMA total, 24 cases; LMS total, 32 cases (Hayashi et al. 2006). In addition, immunohistochemistry showed marked LMP2 expression in cervical epithelial adenocarcinoma tissues as well as cell lines treated with IFN-γ

The defect was localized to Janus-activated kinase 1 (JAK1) activation, which acts upstream in the IFN-γ signal pathway since IFN-γ treatment could not strongly induce JAK1 kinase activity in human uterine LMS cell lines. Sequence analysis demonstrated that the loss of IFN-γ responsiveness in the human uterine LMS cell line was attributable to the inadequate kinase activity of JAK1 due to a G781E mutation in the ATP-binding region (Hayashi et al. 2006).

## Mutations in IFN-γ Signalling Pathway in Human LMS Tissues

**Figure 2:**
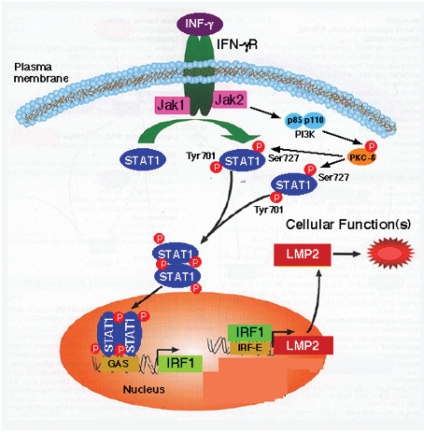
The interferon-γ signaling pathway and mutations in its components found in human uterine leiomyosarcoma. After binding of interferon-γ (IFN-γ) to the type II IFN receptor, Janus activated kinase 1 (JAK1) and JAK2 are activated and phosphorylate STAT1 (signal transducer and activator of transcription 1) on the tyrosine residue at position 701 (Tyr701). The tyrosine-phosphorylated form of STAT1 forms homodimers that translocate to the nucleus and bind GAS (IFN-γ-activated site) elements, which are present in the promoters of IFN-γ-regulated genes. The IFN-γ-activated JAKs also regulate, through as-yet-unknown intermediates, activation of the catalytic subunit (p110) of phosphatidylinositol 3-kinase (PI3K). The activation of PI3K ultimately results in downstream activation of protein kinase C-δ (PKC-δ), which in turm regulates phosphorylation of STAT1 on the serine residue at position 727 (Ser727). The phosphorylation of Ser727 is not essential for the translocation of STAT1 to the nucleus or for the binding of STAT1 to DNA, but it is required for full transcriptional activation. IFNGR1, IFN-γ receptor subunit 1; IFNGR2, IFN-γ receptor subunit 2. Investigation of human uterine LMS tissues (total of 14 cases of LMS tissue sections and normal tissue sections located in same tissue) for somatic mutations in the IFN-γ signal cascade, JAK1, JAK2, STAT1 and Lmp2 promoter region.

IFN-γ treatment markedly increased the expression of LMP2, a subunit of the proteasome, which alters the proteolytic specificity of proteasomes. After binding of IFN-γ to the type II IFN receptor, JAK1 and JAK2 are activated and phosphorylate the signal transducer and activator of transcription 1(STAT1) on the tyrosine residue at position 701 (Tyr701) and the serine residue at position 727 (Ser727) (Parmar and Platanias, 2005; Platanias, 2005) (Fig. 2). The phosphorylated STAT1 forms homodimers that translocate to the nucleus and bind GAS (IFN-γ-activated site) elements in the promoters of IFN-γ-regulated genes (Parmar and Platanias, 2005; Platanias, 2005) (Fig. 2).

Genetic alterations in tyrosine kinases have previously been firmly implicated in tumorgenesis, but only a few serine/threonine kinases are known to be mutated in human cancers (Futreal et al. 2004; Jones and Baylin, 2007; Lengyel et al. 2007; Pajares et al. 2007). For instance, mice carring homozygous deletion of Pten alleles developed widespread smooth muscle cell hyperplasia and abdominal leiomyosarcomas (Hernando et al. 2007), and JUN oncogene amplification and overexpression block adipocytic differentiation in highly aggressive sarcomas (Mariani et al. 2007). Most frequently, leiomyosarcomas have appeared in the uterus, retroperitoneum or extremities, and although histologically indistinguishable, they have different clinical courses and chemotherapeutic responses. The molecular basis for these differences remains unclear. Therefore, the examination of human uterine LMS tissues (32 LMS tissue sections and normal tissue sections located in the same tissue) was performed to detect somatic (tumor-specific) mutations in the IFN-γ signal cascade. The genetic approach has already addressed that somatic mutations in JAK1 molecule correlate to the initiation of several cancer progressions and other disorders (Briscoe et al. 1996; Rossi et al. 2005; Haan et al. 2008). Overall, nearly 42.9% (6/14) of uterine LMS tissues had mutations in the ATP-binding region or kinase-active site of JAK1. Furthermore the genetic approach has already revealed that somatic mutations in the LMP2 molecule or its enhancer region correlate to the initiation of several cancer progressions and other disorders (Heward et al. 1999; Satoh et al. 2006; Krämer et al. 2007; Liu et al. 2007; Mehta et al. 2007), 35.7% (5/14) of uterine LMS tissues had somatic mutations in the Lmp2 promoter region, which is required for transcriptional activation. In addition, genetic examination has already demonstrated that somatic mutations in the STAT1 molecule correlate to the initiation of several disorders (Dupuis et al. 2001; Dupuis et al. 2003; Chapgier et al. 2006; Scarzello et al. 2007). Nearly 35.7% (5/14) of uterine LMS tissues had mutations in the STAT1 intermolecular region. Although the genetic approach has already addressed that marked JAK2 activation causes myelo- and lymphoproliferative disease (Baxter et al. 2005; Campbell et al. 2005; James et al. 2005; Kralovics et al. 2005; Tefferi et al. 2005), no somatic mutation in the ATP-binding region and kinase-active site of JAK2 was detected in uterine LMS. In a recent report, high-resolution genome-wide array comparative genomic hybrodization (CGH) analysis of LMS cases gave gene-level information about the amplified and deleted regions that may play a role in the development and progression of human uterine LMS. Among the most intriguing genes, whose copy number sequence was revealed by CGH, were loss of JAK1 (1p31~p32) and LMP2 (6p21.3) (Lee et al. 2006; Svarvar et al. 2006). The discovery of these mutational defects in a key cell-signalling pathway may be an important development in the pathogenesis of human uterine LMS.

It is probable that the list of new elements involved in IFNs-mediated signalling will continue to grow during the next few years, whereas the contributions of known pathways might need to be re-evaluated. At present, it seems that the activation of more than one signalling pathway is required for the generation of different biological properties of IFNs, and no signalling cascade alone is sufficient for the generation of any given biological end-point. For example, the biological functions of the STAT-, NF-κB and p38 signalling pathways are required for antiviral effects or anti-tumor effects of IFNs, but activation of these pathways alone is not sufficient to elicit an antiviral or anti-tumor response (Ramana et al. 2002; Sizemore et al. 2004). Such a requirement for multiple signalling pathways also seems to be the case for IFNs-dependent anti-proliferative responses, and might reflect the synergistics effects of various signals at the levels of gene transcription and translation; therefore, additional genetic analysis is required to completely elucidate the mutational activation of a key cell-signalling pathway in human uterine LMS.

## Potential Role of Anti-Oncogenic Function by LMP2

The growth of cell lines with JAK1 kinase activity is strongly inhibited by IFN-γ treatment, whereas the growth of JAK1-deficient cell lines is unaffected (Sexl et al. 2003). Similarly, the cell cycle distribution pattern of freshly explanted tumor cells derived from Jak1-deficient tumors shows no response to IFN-γ treatment (Sexl et al. 2003). The growth of the original SKN cells, which had defective JAK1 activity, was unaffected by IFN-γ treatment (population doubling time (PDT) = 15.2 hrs) (Hayashi et al. 2006). In contrast, the growth of JAK1-transfected SKN cells, which had strong exogenous JAK1 activity, was prevented by IFN-γ treatment (PDT = 18.1 hrs). Interestingly, analysis of LMP2-transfected SKN cells showed that exogenous LMP2 expression resulted in cell growth arrest (PDT = 17.9 hrs) (Hayashi et al. 2006). Conversely, the growth of LMP2-transfected SKN cells was unaffected by IFN-γ treatment (PDT = 18.0 hrs). In SKN-Lmp2 transfectants, there is a correlation between the levels of exogenous LMP2 expression and the degree of suppression of the transformed phenotype. The biological function of LMP2 with revertant-inducing activity on SKN cells has been demonstrated.

**Figure 3:**
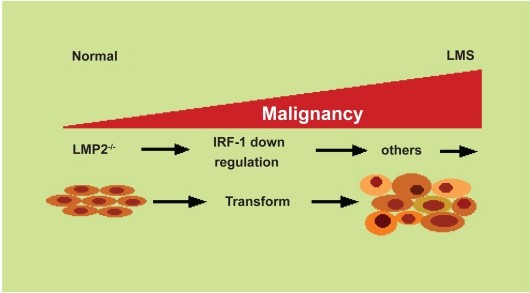
Model for the initiation of uterine leiomyosarcoma tumorgenesis. The initiation of uterine leimyosarcoma development is attributed to defect in LMP2 expression, which results in marked cell proliferation.

Microarray analysis provides insight into the gene expression changes associated with malignant transformation. To investigate whether stable LMP2 expression contributes to cell growth phenotype in SKN cells, the experiment (using Affymetrix human GeneChip HG U133 Plus2.0) demonstrated the expression profile of SKN cells transfected with plasmid without insert (pCEP9) compared with LMP2 coding DNA (pCEP9-LMP2). Microarray analysis has elucidated that LMP2 expression dramatically influences the expression pattern of cell-cycle regulators, especially anti-oncogenic factor interferon regulatory factor 1 (IRF-1), which directly correlate to progressively worsen with the increasing stage and grade of the tumor (Fig. 3).

The down-regulation of MHC expression, including the *lmp2* gene, is one of the biological mechanisms that tumor cells use to evade host immune surveillance (Swann and Smyth, 2007). Recently, the incidence of IFN-γ unresponsiveness in human tumors was examined in several cancers, and revealed that around 33% of each group exhibited a reduction in IFN-γ sensitivity (Kaplan et al. 1998). Nevertheless, LMP2 expression, rather than providing an escape from immune surveillance, seems to play an important role in the negative regulation of uterine LMS cell growth. Defective LMP2 expression is likely to be a risk factor for the development of human uterine LMS, as it is in LMP2-deficient mice.

## Conclusion

To improve the prognosis of human uterine LMS, research experiments were performed to identify the key role of pro- or anti-oncogenic factors that have an important function in their pathogenesis and that could serve as molecular targets for tumor treatment. For this purpose, several research facilities conducted a microarray procedure between human uterine LMS and normal USM and showed that several known pro-oncogenic factors, such as brain-specific polypeptide PEP-19 and c-kit, may be associated with the pathogenesis of human uterine LMS (Kanamori et al. 2003; Wang et al. 2003; Ylisaukko-oja et al. 2006). However, in terms of the tumorgenesis of human uterine LMS, merely comparing the expression of potential pro-oncogenic factors between normal and malignant tissues is not sufficient because the results obtained may be the consequence of malignant transformation and, therefore, not necessarily the cause.

For almost all types of cancer studied to date, it seems as if the transition from a normal, healthy cell to a cancer cell is a step-wise progression that requires genetic changes in several different oncogenes and tumor suppressors. In order to generate a cancer cell, a series of mutations must occur in the same cell. Since the likelihood of any gene becoming mutated is very low, it stands to reason that the chance of several different mutations occuring in the same cell is highly unlikely. For this reason, cells in an elderly body have had more time to accumulate the changes needed to form cancer cells, whereas those in a child are much less likely to have acquired the requisite genetic changes. Importantly, clinical studies have revealed loss of the ability to induce LMP2 expression in human uterine LMS tissues in comparison with normal USM tissues. The discovery of somatic mutational defects in the IFN-γ-signalling pathway may be important for the initial development of uterine LMS. It is noteworthy that stable LMP2 expression contributes to cell proliferation, which directly correlates to the progressive deterioration with increasing stage and grade of the tumor. Recent advances in our understanding of the biology of uterine LMS have concentrated on the impaired IFN-γ signalling pathway. It is clear that mutations in key regulatory genes (tumor suppressors and proto-oncogenes) alter the behavior of cells and can potentially lead to the unregulated growth seen in cancer. Therefore, continued improvement of our knowledge of the molecular biology of uterine LMS may ultimately lead to novel therapies and improved outcome.
